# Evaluation of WIC Online Ordering during the COVID-19 Pandemic: Evidence from an Oklahoma Grocery Store Chain

**DOI:** 10.3390/nu15030478

**Published:** 2023-01-17

**Authors:** Qi Zhang, Junzhou Zhang, Kayoung Park, Chuanyi Tang

**Affiliations:** 1 School of Community and Environmental Health, Old Dominion University, Norfolk, VA 23529, USA; 2 Department of Marketing, Montclair State University, Montclair, NJ 07043, USA; 3 Department of Mathematics and Statistics, Old Dominion University, Norfolk, VA 23529, USA; 4 Department of Marketing, Old Dominion University, Norfolk, VA 23529, USA

**Keywords:** WIC, online ordering, COVID-19, benefit redemption, grocery store

## Abstract

The COVID-19 pandemic is worsening the disparities in food access in the United States. As consumers have been increasingly using grocery online ordering services to limit their exposure to the COVID-19 virus, participants of federal nutrition assistance programs lack the online benefit redemption option. With the support of the US Department of Agriculture (USDA), retailers are pilot-testing online food benefit ordering in the Special Supplemental Nutrition Program for Women, Infants, and Children (WIC). By combining the Oklahoma WIC administrative data, the online ordering data from a grocery store chain in Oklahoma, and the COVID-19 data in Oklahoma, this study examines how WIC participants responded to the online food benefit ordering option and how their adoption of online ordering was associated with the COVID-19 incidence. Results show that from July to December 2020, 15,171 WIC households redeemed WIC benefits at an Oklahoma chain store, but only 819 of them adopted online ordering. They together completed 102,227 online orders, which accounted for 2.7% of the store visits and 2.6% of the monetary value of WIC redemptions at these stores. There was no significant relationship between WIC online ordering adoption and COVID-19 incidence in Oklahoma.

## 1. Introduction

The COVID-19 pandemic is worsening the disparities in food access and increasing food insecurity in the United States (US) [1,2]. Recent research has consistently shown that the pandemic has not only exacerbated the pre-existing disparities but also created new food-insecure households [3,4]. The vulnerable food supply chains, imposed social isolation due to the pandemic, and weak economic and policy interventions together rendered conventional food access solutions inadequate to serve consumers, particularly lower-income consumers [3,5]. For example, food insecurity has been significantly worsened among SNAP participants since the pandemic, which is evidenced by the high rates of food bank use by SNAP participants in 2020 [6]. To cope with the food access challenges brought by the pandemic, some consumers have changed their food purchasing behaviors. For example, consumers use online grocery shopping more frequently, and price and nutrition become less important in food buying decisions [7].

As US consumers have been increasingly using grocery online ordering services to limit their exposure to the COVID-19 virus [8,9], some participants in the federal nutrition assistance programs are seeking the same privilege to redeem their food benefits online. For example, the US Department of Agriculture (USDA) expanded its online purchasing pilot program in the Supplemental Nutrition Assistance Program (SNAP) to 47 states during the pandemic, which allows SNAP online ordering and payment [10]. However, online ordering and payment have not been an option for participants in the Special Supplemental Nutrition Program for Women, Infants, and Children (WIC), although the WIC program served approximately 6.4 million income-eligible pregnant, breastfeeding, postpartum women, infants, and children under age 5 in 2019 [11]. The USDA regulations require that WIC food benefit redemptions must occur in the presence of a cashier [12]. Consequently, WIC electronic benefit transfer (EBT) cards cannot be accepted as an online payment method for WIC-eligible foods. WIC participants can only redeem their prescribed food benefits in-store with WIC-authorized vendors [13,14].

To address this significant issue, in November 2020, the USDA made a USD 2.5 million grant award to pilot test WIC online ordering projects [15]. Moreover, the Consolidated Appropriations Act of 2021 required the USDA to establish a task force to examine alternative ordering and delivery methods in the WIC program, including online ordering [16]. The American Rescue Plan Act of 2021 allocated USD 390 million for WIC modernization, program innovation, and outreach [17]. Therefore, research on WIC online ordering is vital for WIC policymakers as they seek to explore new WIC benefit delivery methods.

Since WIC is a federally funded but state-operated program, some state agencies have tested their own “online ordering” models under the current legislation, i.e., only ordering the food online but not using the WIC EBT card to make the payment online [18,19]. A grocery chain in Oklahoma, anonymously referred to as the XYZ store, developed an application (app) during the pandemic that allowed shoppers to order food online and pick it up in-store or curbside later with their WIC EBT card payment. XYZ is the only store chain that provides online ordering access for WIC participants to redeem their food benefits in Oklahoma. An evaluation of the online ordering activities in XYZ stores can demonstrate how WIC participants responded to the availability of the online ordering option during the pandemic. The results are important for the USDA and WIC state agencies as they prioritize the development and implementation of WIC online ordering during and after the pandemic [20]. Given the significant knowledge gap regarding how WIC participants might use online ordering, this study aimed to examine its adoption in one grocery chain within one state and hypothesized that COVID-19 incidence was associated with the adoption.

## 2. Methods

### 2.1. Setting

XYZ is a “WIC-only” store chain, which means all food stocks in the store are WIC-eligible, and all or nearly all of their revenues are from WIC [21]. XYZ has 10 stores in Oklahoma, which serve approximately 30% of the state’s WIC participants and take in approximately one-fourth of the total WIC redemption dollars in Oklahoma. XYZ store developed an online ordering system in the first half of 2020, pilot tested it in several stores, and fully implemented the system in July 2020. Any customers can use the store-developed app, XYZ app, to order food in a designated XYZ store without any online payment requirement, using only their names and cell phone numbers for identification and communication purposes. When the staff at the designated store receives an order from the store system, they pack the ordered food for pick up. Since the current WIC regulation does not allow online payment with WIC EBT cards, the XYZ app did not have an online payment function. Customers could pay for the order curbside or in-store with their WIC EBT cards or other forms of payment, e.g., cash, credit, or debit cards. The XYZ app was available both in iOS and Android systems and could be downloaded from Apple App Store or Google Play.

### 2.2. Data

The analytical data in this cross-sectional study included the WIC administrative data from the Oklahoma WIC agency, the online ordering data from the XYZ stores, and the COVID-19 data from the Oklahoma State Department of Health [22]. The WIC administrative data include the participating households’ socio-demographics, household members’ WIC participation status, WIC food benefit prescriptions, and redemption records in all WIC-authorized stores in Oklahoma. The online ordering data set contains information about individual transactions, such as the transaction date, the description of each food item, the number of units redeemed, and the monetary value redeemed. The online ordering data have timestamps to record when a customer placed an online order, the scheduled pick-up time, the transaction information (e.g., the food items ordered and finally transacted), price, and units of food items. The WIC administrative data were matched with the online ordering data by the WIC household identification number and were merged with the COVID-19 data based on the residential county of the household and the week of the transaction. Since redemption behavior is associated with the household instead of individual participants, the unit of analysis is the OK-WIC-XYZ household that participated in OK (Oklahoma) WIC and redeemed WIC benefits in XYZ stores at least once. This is the first store chain-level data set in the nation to have its WIC online ordering information, individual WIC redemption transaction records, and the corresponding COVID-19 incidence at the county level combined into a single set of information.

Since Oklahoma declared the COVID-19 emergency statewide on 15 March 2020 [23], the overall study period was from 3 February 2020 to 3 January 2021, focusing on capturing weekly data. The online ordering study period was from 1 July 2020 to 31 December 2020, since online ordering was fully implemented in XYZ stores in July 2020. To examine the trend of WIC redemption, the weekly number of orders was calculated for the 48 weeks of the overall study period. The four-week moving average of COVID-19 incidence was also calculated.

### 2.3. Measure

To measure WIC households’ overall redemption behaviors, the number of weekly WIC redemption store visits was used, which was defined as the total number of redemption visits to any WIC-authorized store per household per week. Two similar measures were created to capture the number of weekly WIC redemption visits to XYZ stores and the weekly number of online orders among OK-WIC-XYZ households. To differentiate the first-time online ordering shoppers and returning shoppers (i.e., those using online ordering two or more times), the numbers of weekly online orders were also calculated separately for these two groups.

To capture participants’ online ordering choices, a binary indicator was created to measure whether a WIC household was engaged in online ordering or in-store shopping during a certain week. The primary explanatory variable was the moving-average COVID-19 incidence that week within the county. The control variables included the household’s race, which was determined by the eldest WIC participant in the household, whether there were infant, child, or woman participants in the household, the number of WIC participants in the household, the household size, the households’ annual income, and the population of the household’s residential county in 2019 [24].

### 2.4. Data Analyses

Descriptive statistics were estimated for the socio-demographics of in-store customers vs. online ordering customers in XYZ stores. The differences between the two groups were tested with chi-square and Wilcoxon rank-sum tests. Ordinary least squares (OLS) regression was applied to examine the relationship between the weekly WIC redemption visits and the weekly moving average COVID-19 incidence. Random-effects logistic regression models were employed to examine the adoption of online ordering in a certain week among all OK-WIC-XYZ households given the COVID-19 incidence while controlling socio-demographics to address the potential biases in WIC redemptions across time. The natural logarithm of the COVID-19 incidence was used in the logistic regression models due to skewed incidence distributions. A value of *p* < 0.05 was treated as statistical significance. Stata 15 was employed to conduct the statistical analyses [25]. This study was approved by the Institutional Review Board at Old Dominion University.

## 3. Results

In the study period, 60,998 WIC households redeemed their WIC benefits via 933,762 store visits in Oklahoma. Among them, 29.7% of these WIC households had ever redeemed WIC benefits at XYZ stores in 2020, constituting 152,873 of all store visits and accounting for 25.1% of the monetary value of WIC redemptions in Oklahoma. In the online ordering study period, i.e., the last six months of 2020, 15,171 WIC households redeemed WIC benefits at XYZ stores via 83,137 XYZ store visits. Still, only 819 WIC households adopted online ordering. They together completed 2227 online orders, which accounted for 2.7% of XYZ stores visits and 2.6% of the monetary value of WIC redemptions at XYZ stores.

**Figure 1:**
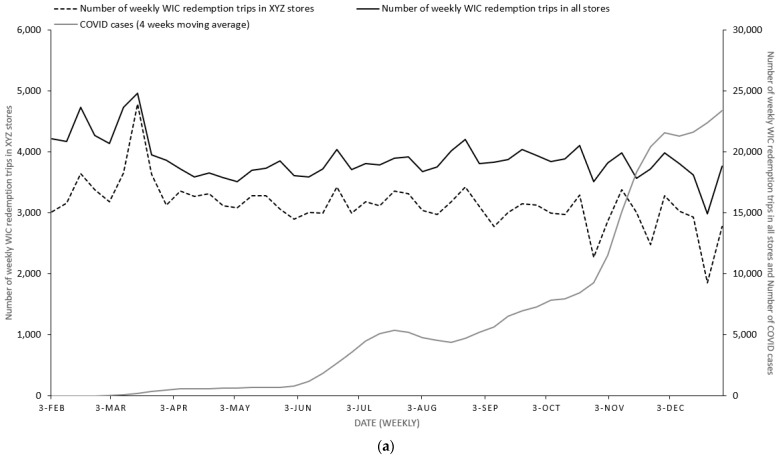

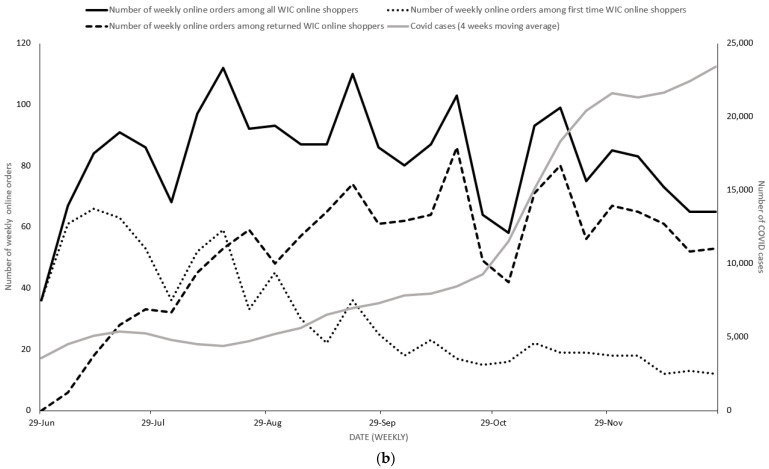
(**a**) The trends in the number of weekly WIC redemption visits in all stores and in the XYZ stores and the COVID-19 incidence. (**b**) The trends of weekly online orders and the weekly COVID-19 incidence.

Figure 1a illustrates the trends in the number of weekly WIC redemption visits in all Oklahoma stores and specifically in XYZ stores, as well as COVID-19 incidence. In general, the two curves of WIC redemption visits resemble each other during the study period. For both curves, there was a clear spike in the middle of March when Oklahoma declared a COVID-19 emergency, suggesting WIC households increased their redemptions around that time. As the COVID-19 incidence significantly increased over time until October, the trends were relatively stable. However, when the COVID-19 incidence increased still more significantly in the last two months, there was a downward trend in both curves. The simple linear regression results indicated a significantly negative relationship between the number of WIC redemption visits in all stores and in XYZ stores and COVID-19 incidence (b = −0.078, *p* = 0.018 for all store redemption visits; b = −0.027, *p* < 0.001 for XYZ store redemption visits).

Figure 1b depicts the trends of weekly online orders with the weekly COVID-19 incidence. The number of WIC online orders increased to a peak in August, one month after online ordering was fully implemented in July 2020. Then the total online order numbers remained stable over time. Since the number of weeks in the study period is relatively small (26 weeks), no linear regression was conducted to examine the relationship between the number of weekly online orders and COVID-19 incidence. It is worth noting that the number of online orders among first-time users of online ordering reached a peak in the first month and then showed a waning trend over time. The number of weekly online orders among returning WIC online order patrons increased gradually over time until reaching a peak in October 2020, which indicates that online ordering was losing returning customers.

Table 1 presents the socio-demographics of the study sample in the online ordering period and compares them between in-store and online ordering customers. There was a significant difference in racial/ethnic composition between online ordering and in-store customers (*p* < 0.001). The percentage of non-Hispanic Whites among all WIC online order customers (25.2%) was double that among all in-store patrons (12.6%). Non-Hispanic Black and Hispanic customers accounted for a smaller percentage of those using WIC online ordering (12.8% versus 50.7%, respectively) compared with their percentage among WIC in-store customers (16.7% versus 58.3%, respectively). The proportions of households with infant and with women participants among the WIC online ordering customers were 42.4% and 36.4%, respectively, significantly higher than among the WIC in-store customers (33.9% and 32.7%, respectively) (*p* < 0.001 and *p* = 0.032, respectively). Households with a child participant did not show a significant difference between WIC in-store customers and online ordering customers (*p* = 0.27). The WIC online ordering customers had a higher percentage of multi-participant households than in-store customers (*p* < 0.001), and the mean household size of online ordering customers was significantly smaller than that of in-store customers (3.90 vs. 4.09, respectively, *p* < 0.001). In addition, the WIC online ordering customers had a higher mean income than the in-store customers (USD 23,330.71 vs. USD 21,834.03, respectively, *p* = 0.017).

Table 2 presents the results of the logistic regression of the individual household’s online ordering adoption on COVID-19 incidence while controlling socio-demographics. COVID-19 incidence did not significantly impact WIC participants’ likelihood to adopt online ordering (OR = 0.962; 95% CI = 0.865, 1.070; *p* = 0.475). Compared with non-Hispanic Whites, all minority groups’ ORs were significantly smaller than 1 (*p* < 0.001), which indicates minority households were less likely to adopt WIC online ordering. Households with infants were more likely to order online in XYZ stores (OR = 1.369; 95% CI = 1.001, 1.871; *p* = 0.049), while households with or without a woman or child participant did not show statistical difference (*p* > 0.05). Number of WIC participants in the household was positively associated with the likelihood of adopting WIC online ordering (OR = 1.440; 95% CI = 1.074, 1.929; *p* = 0.015 for households with two WIC participants; OR = 1.760; 95% CI = 1.006, 3.078; *p* = 0.048 for households with three or more WIC participants). In addition, larger households had a lower OR to adopt online ordering (OR = 0.811; 95% CI = 0.748, 0.878; *p* < 0.001), while household income was marginally significantly related to the adoption of WIC online ordering (*p* = 0.056). The residential county population was negatively associated with the likelihood of WIC online shopping (OR = 0.581; 95% CI = 0.469, 0.718; *p* < 0.001).

## 4. Discussion

This study examines the adoption of online ordering in a WIC-only store chain, and the results offer much-needed evidence for WIC stakeholders who are considering how to modernize the WIC program as required by the American Rescue Plan Act of 2021 [17]. As a secondary data analysis, this study serves as a basis for future interdisciplinary research (e.g., sociology, psychology, and anthropology) or a multi-methods approach (e.g., survey and experiments). The results achieved to date indicate that minority participants were less likely to adopt the new redemption method compared with non-Hispanic White peers, which indicates that more efforts are needed to equalize access to and adoption of WIC online ordering across races/ethnicities [26,27]. WIC households with infants were more likely to adopt WIC online ordering, suggesting a behavioral advantage of curbside payment in the WIC online ordering model, i.e., participants do not need to leave the car to receive the order. Households with more participants were more likely to adopt online ordering, suggesting that WIC online ordering may attract households with larger WIC benefit balances who need to redeem a lump sum of benefits. However, it is possible that if the household is too big participants may not be motivated to adopt WIC online ordering, since they may visit other grocery stores for general grocery shopping, not purely for WIC benefit redemptions.

Although it is highly expected that WIC online ordering will benefit WIC customers’ redemption experiences [15], this study observed a low adoption rate among XYZ customers. Although the study itself did not provide evidence to explain the low adoption rate, we conducted an additional qualitative study whose results are summarized in another manuscript. We propose three theories to explain the results that will be important to explore in future research.

First, the “COVID-19 pandemic” proposition. The pandemic has changed consumers’ buying behaviors, such as stockpiling and reduced numbers of shopping visits [28,29,30]. The trend analyses indicate that the number of redemption visits in XYZ stores was negatively associated with COVID-19 incidence. However, COVID-19 incidences were not significantly related to the adoption of online ordering, which suggests that WIC consumers might not be concerned enough about COVID-19 to adopt online ordering.

Second, the “crossing the chasm” proposition. A traditional technology adoption life cycle is a bell-shaped curve representing the groups of consumers adopting new technology, progressing from innovators, early adopters (visionaries), early majority (pragmatists), late majority, to finally, laggards [31]. However, a large gap, or a chasm, often shows up in the adoption rate between the early adopters and the early majority [32]. Chasm is common in the diffusion of new technology, and many innovations have failed when trying to make it across this divide [32]. The USDA has invested in technology to better serve WIC participants remotely during the pandemic, but more research and efforts are needed to ensure that these technology innovations will cross the adoption chasm [26]. Although previous studies consistently found that consumers are more likely to shop online during the pandemic and prospective interviews indicated that WIC participants might be interested in WIC online ordering [18,33], the preliminary results from XYZ stores indicate that a potential chasm might exist in WIC online ordering adoption. Online ordering was not able to attract the large number of WIC consumers that had been anticipated.

Traditional diffusion theories suggest that innovators and early adopters are mainly influenced by mass-media communications, while the early majority (imitators) is mainly affected by interpersonal communications (e.g., word of mouth) [34]. Although the pandemic might have had a negative impact on word of mouth since it imposed social isolation, other factors may have contributed to the low word-of-mouth communication among WIC participants. For example, Paley et al. (2019) suggested that low-income consumers tend to be less active in word-of-mouth information sharing than higher-income consumers since the intended purchasing behaviors remind them of the limited wealth they have [35]. Thus, to attract more WIC participants, retail stores and WIC agencies may need to switch the focus of the promotion programs from mass communication to interpersonal communications, such as a peer reference program or cell phone messages.

Moreover, online ordering has to prove its value and showcase the successes of consumers who have adopted online ordering, such as being convenient, saving time, and reducing the risk of COVID-19 infection [33,36]. However, SNAP participants were not very interested in ordering groceries online due to delivery costs, low selection control (e.g., unable to pick their favorite produce), and lack of hedonic value (e.g., joyfulness) in online shopping [37,38]. Compared with their higher-income counterparts, SNAP participants were less likely to shop online due to several factors, e.g., lack of online shopping knowledge and preference for in-store shopping [38,39], which could be applicable to WIC participants as well. WIC agencies and vendors may think about how to nudge WIC participants to try online ordering and experience the value of this service.

Third, the “alternative shopping method” proposition. Compared with the default in-store shopping method, online ordering provides an alternative shopping method [33]. The online ordering and store pickup model needs to compete with the existing in-store shopping method to win over customers. The reduced risk of COVID-19 infection in online ordering, for instance, could nudge WIC some customers to change their shopping behaviors [36]. Moreover, the convenience of curb-side pick-up and time saving could attract customers, particularly those who need to shop with children or have a busy working schedule. However, since customers may visit XYZ stores a limited number of times in a month, these advantages may not be overwhelming compared with in-store shopping, in which customers have more control over food selection, e.g., fresh produce [38]. The low adoption rate of online shopping may reflect the strong advantage of the current in-store shopping model compared with the preliminary online ordering model, which also explains why the online ordering model is losing returning customers.

The WIC online ordering model examined in this study was in its early stage; it did not offer online payment and home delivery. Moreover, the XYZ app did not achieve full functionality. For example, it was not linked to users’ WIC benefit records. Since online redemption has not been widely developed in the WIC program, its full benefits have not been achieved, which may play a role in reducing its adoption among WIC customers. Previous studies have shown a significant improvement in WIC benefit redemption with the WIC app [40,41]. By bundling the XYZ app with the existing WIC apps, WIC participants would be able to see their remaining benefits easily while ordering online.

## 5. Limitations

These results need to be interpreted carefully, with due acknowledgment of their limitations. First, given the data limitations, this study cannot identify other confounding factors affecting customers’ redemption behaviors, e.g., individual customers’ COVID-19 status. Moreover, this time-sensitive study was conducted in a limited observation time period. Finally, this study is based on short-term individual store-chain data from a WIC-only store, which may limit the generalizability of the findings to other store types, such as supermarkets or convenience stores. This limitation should be addressed with future explorations conducted in more diverse shopping settings. Despite the above limitations, this study provides important preliminary evidence to policymakers, WIC-authorized vendors, and researchers eager to examine the adoption of WIC online ordering during and after the COVID-19 pandemic.

## 6. Conclusions

No significant relationship was identified between WIC online ordering adoption and COVID-19 incidence in 2020. However, minority participants had a lower likelihood of adopting WIC online ordering compared with non-Hispanic White peers. Households with infants and more participants were more likely to adopt WIC online ordering. The initial low online adoption rates deserve further studies to understand the facilitators and barriers for WIC participants to take advantage of this new shopping option. Future interdisciplinary, multi-method research can help improve WIC online ordering to facilitate participants’ redemption experience.

## Figures and Tables

**Table 1:** Descriptive statistics of the socio-demographics between WIC XYZ in-store shoppers and online shoppers.

Variable	All N = 15,171(%)	WIC in-Store XYZ Shoppers *n* * = 14,352(%)	WIC Online Shoppers *n* * = 819(%)	p **
Racial/ethnic group				<0.001
Non-Hispanic White	2008 (13.2)	1802 (12.6)	206 (25.2)	
Non-Hispanic Black	2495 (16.4)	2390 (16.7)	105 (12.8)	
Hispanic	8783 (57.9)	8368 (58.3)	415 (50.7)	
Others	1885 (12.4)	1792 (12.5)	93 (11.4)	
Did the household have an infant participant?				<0.001
Yes	5209 (34.3)	4862 (33.9)	347 (42.4)	
No	9962 (65.7)	9490 (66.1)	472 (57.6)	
Did the household have a child participant?				0.27
Yes	10,774 (71.0)	10,178 (70.9)	596 (72.8)	
No	4397 (29.0)	4174 (29.1)	223 (27.2)	
Did the household have a woman participant?				0.032
Yes	4991 (32.9)	4693 (32.7)	298 (36.4)	
No	10,180 (67.1)	9659 (67.3)	521 (63.6)	
Number of WIC participants				<0.001
1	8999 (59.3)	8604 (59.9)	395 (48.2)	
2	4561 (30.1)	4272 (29.8)	289 (35.3)	
≥3	1611 (10.6)	1476 (10.3)	135 (16.5)	
Household size (people)	4.08 (1.59)	4.09 (1.59)	3.90 (1.62)	<0.001
Household annual income (USD)	21,914.82 (14,012.56)	21,834.03 (13,936.97)	23,330.71 (15,216.27)	0.017

* Statistics presented: *n* (%); mean (SD); ** Statistical tests performed: chi-square test of independence; Wilcoxon rank-sum test.

**Table 2:** Regression results of the random-effects model of weekly WIC online ordering adoptions among XYZ shoppers.

	Using Online Ordering during a Week? (Yes/No)		
	Odds Ratio	95% CI	*p*
Racial/ethnic group			
Non-Hispanic White	Ref		
Non-Hispanic Black	0.223	0.151, 0.329	<0.001
Hispanic	0.226	0.169, 0.304	<0.001
Others	0.286	0.197, 0.415	<0.001
Did the household have an infant participant?			
No	Ref		
Yes	1.369	1.001, 1.871	0.049
Did the household have a child participant?			
No	Ref		
Yes	1.302	0.880, 1.925	0.187
Did the household have a woman participant?			
No	Ref		
Yes	0.946	0.684, 1.308	0.735
Number of WIC participants			
1	Ref		
2	1.440	1.074, 1.929	0.015
≥3	1.760	1.006, 3.078	0.048
Household size	0.811	0.748, 0.878	<0.001
Log (Income)	1.077	0.998, 1.162	0.056
Log (County COVID-19 cases)	0.962	0.865, 1.070	0.475
Log (County population)	0.581	0.469, 0.718	<0.001
N	80,460		
chi2	290.625		
*p*	<0.001		

## Data Availability

Due to the non-disclosure agreements signed with the Oklahoma State WIC agency and the XYZ store chain, the authors cannot make the WIC redemption data and the online ordering data publicly available.
